# Loss of Subscriptions

**Published:** 1913-02

**Authors:** 


### Loss of Subscriptions

We have lost a regrettably large number of subscribers by death and a few by retirement and removal to parts unknown. Excepting these inevitable losses and the preliminary erasure of a number of nominal subscriptions in arrears for many years, we have lost only 22 subscribers in nearly a year and a half and have added several hundred to the list. What is particularly gratifying, is the steady receipt of orders from distant parts, even outside of the United States, not the result of any campaign nor directly traceable to opportunities of seeing copies in the hands of others. We feel, however, that the BUFFALO MEDICAL JOURNAL has a special field for usefulness in Western New York, as indicated approximately by the Associate Staff, and we request missionary work by present subscribers. This request is justified by the fact that the editorship can scarcely be classified as a "gainful occupation," although the JOURNAL itself is on a firm financial footing; and by the fact that every subscriber is a source of strength, not so much because of the subscription fee, as by his influence and interest.

